# FCC – An automated rule-based processing tool for life science data

**DOI:** 10.1186/1751-0473-8-3

**Published:** 2013-01-11

**Authors:** Simon Barkow-Oesterreicher, Can Türker, Christian Panse

**Affiliations:** 1 Functional Genomics Center Zurich (FGCZ), Swiss Federal Institute of Technology Zurich (ETHZ)|University of Zurich (UZH), CH-8057 Zurich, Winterthurerstrasse 190, Switzerland

**Keywords:** Computing, Automatization, High-throughput, Data processing

## Abstract

**Background:**

Data processing in the bioinformatics field often involves the handling of diverse software programs in one workflow. The field is lacking a set of standards for file formats so that files have to be processed in different ways in order to make them compatible to different analysis programs. The problem is that mass spectrometry vendors at most provide only closed-source Windows libraries to programmatically access their proprietary binary formats. This prohibits the creation of an efficient and unified tool that fits all processing needs of the users. Therefore, researchers are spending a significant amount of time using GUI-based conversion and processing programs. Besides the time needed for manual usage, such programs also can show long running times for processing, because most of them make use of only a single CPU. In particular, algorithms to enhance data quality, e.g. peak picking or deconvolution of spectra, add waiting time for the users.

**Results:**

To automate these processing tasks and let them run continuously without user interaction, we developed the FGCZ Converter Control (FCC) at the Functional Genomics Center Zurich (FGCZ) core facility. The FCC is a rule-based system for automated file processing that reduces the operation of diverse programs to a single configuration task. Using filtering rules for raw data files, the parameters for all tasks can be custom-tailored to the needs of every single researcher and processing can run automatically and efficiently on any number of servers in parallel using all available CPU resources.

**Conclusions:**

FCC has been used intensively at FGCZ for processing more than hundred thousand mass spectrometry raw files so far. Since we know that many other research facilities have similar problems, we would like to report on our tool and the accompanying ideas for an efficient set-up for potential reuse.

## Background

Most bioinformatics workflows involve handling and computationally demanding processing of raw files. In spite of strong efforts from standardization committees like the Proteomics Standards Initiative (PSI) to facilitate community-driven standardization of file formats (see e.g. [1]), conversion into compatible file formats is still a major task in this process. Initial efforts to reverse engineer the proprietary formats of mass spectrometry vendors to access the mass spectra directly are underway (e.g. [2]) but are still working on only very few raw data types. An example for a frequently used file converter is the ProteoWizard msconvert tool [3], which can deal with most of the binary vendor formats (using their provided Windows libraries) and features a graphical user interface as well as a command line interface.

At FGCZ, fourteen different mass spectrometers from three vendors produce between 50 and 100 raw data files per day that have to be dealt with according to the needs of FGCZ users in a reproducible fashion. Mass spectrometer raw files are often produced with different device options, e.g. with different ion fragmentation methods like ETD or CID. Furthermore, different protein quantification methods require special converter options. Lastly, new converter programs are released frequently so that data have to be converted again with the new versions of the executables. An example for a new method is the H-Score algorithm, a rescoring approach for improved peptide identification [4]. The introduction of this postprocessing step has led to a lot of new conversions of already existing proteomics data sets at FGCZ. A manual handling of all these different conversion tasks would be cumbersome and error prone. Another problem comes from the fact that the conversion programs and libraries are only built for a single operating system and sometimes do not offer a graphical user interface. Every user, therefore, would have to maintain both linux and windows operating systems and be command line savvy to be able to make use of all different options.

There exist several integrated systems for proteomics data management, examples are PRISM [5] or B-Fabric [6]. These systems are usually designed for the business use cases of specific research facilities. The FCC system, on the contrary, is designed to be most flexible to adapt to specific uses. It can also operate under the hood of a more general data management system, like we implemented it in our in-house developed B-Fabric system, to offer one way of dealing with processing tasks.

## FCC features

To overcome the described problems and to automate all file processing tasks we have developed the FCC system. We designed it to meet the following requirements: 

•Robustness: FCC should handle unexpected exceptions from executables and from corrupt input files.

•Maintainability: To allow other developers to quickly adapt the source code to their needs, the entire program should consist of less than 500 lines of readable code.

•Simple configuration: There should be only one single configuration file that can be edited in a local directory on any computer. No direct access to the conversion servers should be necessary for the user.

•Full CPU utilization: All available CPU cores of all machines should get used in parallel.

•Commercial software licenses should be used most efficiently for all users.

•Multi platform compatibility: Support for Windows 32Bit, Windows 64Bit, and Linux simultaneously (note that the provided example files are all Windows based).

•Customizability: File path filters for project, instrument, user, time, and regular expression patterns should allow customized conversion tasks.

•Extensibility: New file matching rules, converter programs, and converter server can be added on-the-fly without interruption of the conversion processes.

•Implementation of workflows: FCC should be able to deal with multi-level workflows where the output of one processing can get processed by the next executable.

## Implementation

The design of FCC is inspired by the OpenBSD Stateful Packet Filter [7]. The equivalent of packets are the input file candidates on the shared file system that are compared against the filter rule set. The default is to block processing and let only files pass that match the defined set of rules triggering a configured pass action. When a file matches a rule, it gets forwarded to a wrapper script that packages it together with the execution command of the respective converter and the necessary processing parameters. Correctness and error handling of the conversion process lies in the responsibility of the wrapper code (see Listing 1 as example).

### Listing 1 Example Mirosoft batch script for generating Mascot Generic Files (mgf) using ProteoWizard msconvert and the H-score algorithm written in python

**Figure 1:**
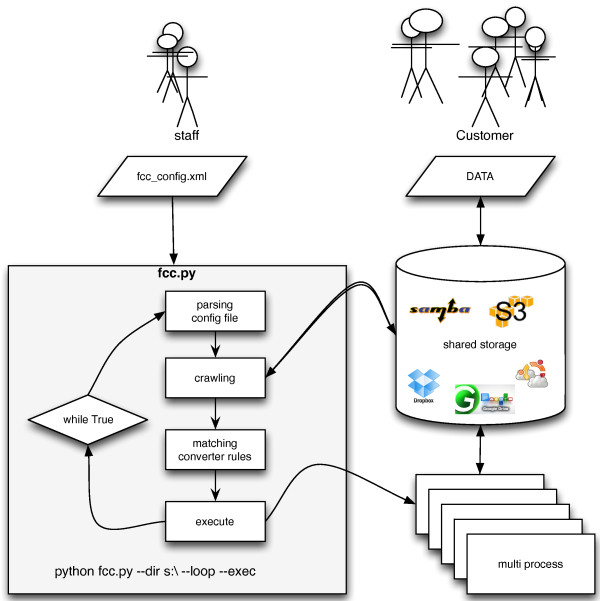
**Flow diagram of FCC.** Flow diagram of the FCC system: 1. parse the configuration file, 2. crawl the shared storage space, 3. match file path filters and converter rules, and 4. execute processing jobs. The customers use mass spectrometers to produce raw data on a shared storage while the bioinformatics staff is responsible for the FCC configuration in a Subversion version control system.

FCC itself consists of a python program and an XML configuration file. The configuration file is divided into two parts: (i) the configuration of the converter programs and (ii) the filtering rules for the candidate files. On every participating server, FCC monitors the shared file system and distributes conversion tasks among all available CPU cores automatically. The servers can run on different operating systems. The following steps are conducted on every converter server (see Figure 1): (1) FCC parses the configuration file. (2) It crawls the specified shared file location creating a complete list of candidate files for conversion in a python data structure. (3) In the next step each file path on that list is tested against the rule set. (4) If a file matches and the output file does not already exist, the processing is triggered. An execution command consisting of the path to the executable, conversion options, and the input and output file paths is fed to the pool of processors on that computer. FCC logs successful as well as erroneous results in a central log file and guarantees that erroneous processes will get excluded in the next iteration to avoid an endless loop of processing attempts of corrupt input files.

Parallelization is implemented with the python multiprocessing.pool class. The commands get executed as soon as resources become available. We use the functionality of the native python class to mimic a minimalistic local resource management system avoiding the complexity of larger systems, like the Oracle Grid Engine. After the conversion is done, the crawling step is started over to look for new files. A more elegant solution than to reiterate would be to use a notification service from the underlying operating system, e.g. the fileSystemWatcher class of Microsoft .NET or the inotify service on linux. The problem is that these services are dependent on the operating system.

**Figure 2:**
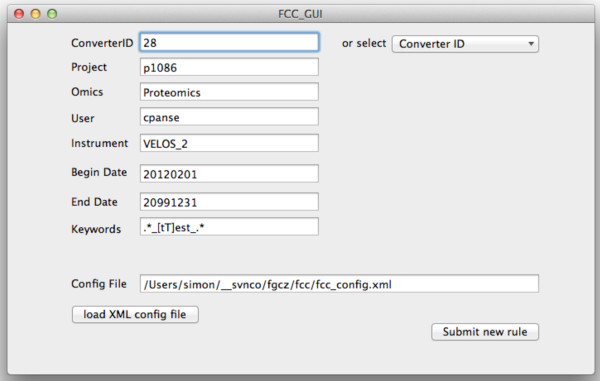
**FCC GUI.** Mac OS X graphical user interface for adding rules to the configuration XML file.

The configuration of rules and converters in the XML config file is normally done using a text editor. In order to reduce the introduction of errors, we have developed a simple mac cocoa application for adding new rules (see Figure 2). This application simplifies configuring new filtering rules and simultaneously checks the validity of the XML file. The configuration file can be distributed using, e.g., the Apache Subversion versioning and revision control system which can be set up to automatically update all participating computers. Choosing Subversion allows to save a history of all configuration files with their specific conversion parameters to be able to reproduce all conversion tasks at a later point in time. Any system of picking up the latest configuration would also be sufficient to allow a remote configuration. Examples would be Dropbox, Google Drive, or an SSH based script.

Listing 2 shows example FCC configurations that include the following parameters:

•converterID: identifier to select the converter in the filtering rules

•converterDir: name of the directory where the output files are saved

•converterCmd: path to the executable of the converter

•converterOptions: command-line parameters for the converter

•fromFileExt: file extension of the input file

•toFileExt: file extension of the output file

•hostname: DNS hostname of the computer where the converter is installed

### Listing 2 Example for FCC processing configuration

If the executables do not need a commercial license, they can be distributed together with the configuration files. If their path is relative to the checkout directory there is no individual setup necessary. Commercial licenses that can be bound to specific Hardware addresses have to be installed individually on every participating server. A setup without commercial licenses, at least for a part of the converter programs, is especially useful in a virtualized computer environment where converter servers can easily be cloned and spawned on demand. Adding an additional converter server to deal with a high load then only means to start an additional virtual machine, alter the hostname, enter the new hostname into a number of converter configuration lines, and commit the configuration file to the Subversion system. New files matching these rules will then get automatically processed on the additional server. A rule-of-thumb for the time point to spawn a new server is a continuous utilization of fifty percent.

To use Subversion, a central repository is needed as well as Subversion clients on all converter servers. On the converter servers a batch script can be set up to automatically update the checkout directories. To be able to use a service like Dropbox or Microsoft SkyDrive, the respective clients have to be set up to use a common directory for all users on all converter servers as well as on the user PCs.

## Results and discussion

In the following, we describe our set-up at FGCZ. We run FCC on five 8-core servers, running Windows 64bit, Windows 32bit, and Linux operating systems. One Windows server is assigned to convert Thermo Velos and Orbitrap raw files, another for AB Sciex Q-TOF and TripleTOF wiff files. The third windows server converts Waters raw files into netCDF files. The fourth Windows server runs different open source converter programs, e.g. for mzXML conversion. The linux server handles in-house developed converter scripts, e.g. for creating exclusion lists.

The mass spectrometer raw files are organized in a project-centric way. We have run about 1200 different projects, yielding 101,000 raw files from fourteen mass spectrometers over the last ten years, 20,000 alone in 2011, approximately 50 per day (see Table 1 for an overview of the current status at the FCGZ). The large number of files makes a consistent and meaningful naming convention and organisation of the file storage indispensable. Given that the folder structure is organized in a meaningful hierarchy, it becomes possible to filter the candidate raw files during the crawling step on the basis of their file paths. The definition of the filtering rules and the configuration of the converters has to be adapted to the individual situation and conventions at other research facilities. If they do not match the set-up of FGCZ, some minor alterations have to be done in the python source code.

**Table 1:** The set-up at FGCZ consists of 14 mass spectrometers and 217 converter rules for 1200 projects


#rules	217
#converters	50
#projects	1200
#instruments	14
#hosts	5

At FGCZ, the shared file system for raw files and processed files is organized as follows: every project has a unique identifier. The folder hierarchy then includes the scientific area (the -omics technology), the name of the instrument that generated the file, the name of the user that measured the sample, and the creation date of the file. At the end the file name can include keywords as description. These keywords can be used for regular expression filtering.

An example filtering rule is shown in Listing 2. This rule triggers the converter with the converterID 28, e.g., for this raw file:

The command on Figure 1 lets FCC run in an endless loop on every converter server. Alterations to the configuration file will become effective in the next iteration without requiring a restart. The raw files get pushed automatically from the mass spectrometer PC to a spool directory on a samba share by an external process running on the instrument PCs. To keep the raw data secure, we created a special active directory user for FCC and grant only him write permissions on the spool directory. After processing is done, the resulting files get automatically synchronized from the spool directory to a user-accessible read-only share by a daemon process on the file server. The raw files reside on the user-accessible archive next to the processed files in case users need them for manual processing. The spool directory gets cleared monthly to prevent long crawling times.

**Figure 3:**
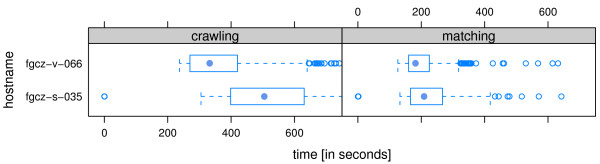
**FCC crawling and matching time.** The graphic shows the average FCC crawling and matching times over a six month period. The times are dependant on the number of file candidates, network protocol, network load, and file system type (local or remote).

**Figure 4:**
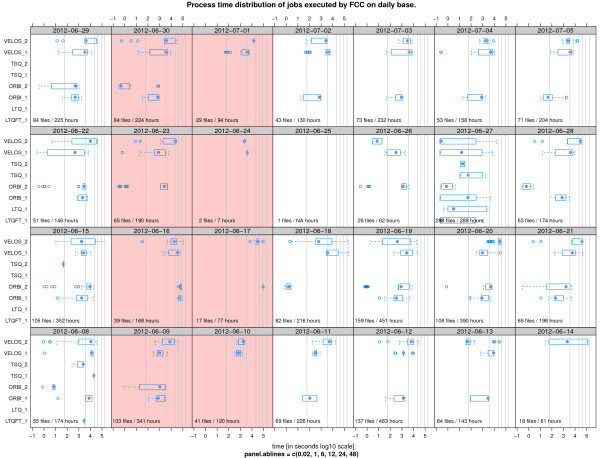
**FCC processing time.** The trellis box-plot graphic shows the distribution of the process time of the file conversions and filtering of different mass spectrometer files. The x-axis is log10 based. The grey lines show the time (1 minute, 1, 6, 12, 24, 48 hours). Each panel shows the data from a particular day. The red boxes are weekend days. The processing time changes unpredictably with the kind of instrument settings, e.g. the measurement gradient time, and with the different conversion, the filtering options of the converter programs.

The FCC configuration at FGCZ defines 217 different converter rules. There is a set of standard converter rules defined for every instrument that deals with all files coming from the specific instruments. Additionally, there are customized rules active for 73 different user projects. We have investigated the FCC crawling and matching time (Figure 3) over a six month period and the processing time for diverse raw files and instruments on a number of exemplary days (Figure 4). The processing times can vary largely. As an example, the average time for the Mascot Distiller processing of Thermo Velos raw files is two hours per file. Although it can spread out to more than 24 hours for exceptional large files (see date 2012-06-14 in Figure 4).

We chose Samba technology for file sharing, because it provides enough data throughput even for very large file sizes and provides all necessary security features for a multi-user environment. On the other hand, it could also be beneficial to use a web-based sharing service, like Amazon S3. This would have the advantage that converter servers can also be set up on another physical location, e.g. in a compute cloud using services like Amazon EC2, although the file size of the mass spectrometry raw files can prohibit such a hybrid set-up.

Since the executables as well as all input and output file extensions are freely configurable, FCC in general could also be used for other workflows where data has to be processed efficiently. An example would be the task of downsampling and further processing of high resolution images making full use of todays multicore computer systems. The custom regular expressions in the filter rules, thereby, allow to match the naming convention of a local file system. For subsequent workflow steps one would set up different rules for processes that take the output files of other processes as input. Although, for more complex workflows it may be advisable to use a dedicated workflow system, like KNIME [8], for example.

For very large set-ups, it could be beneficiary to use a Grid Engine system instead of running the python multiprocessing library on every converter server. This would have the advantage that a single FCC process on the grid engine master server, ideally the file server, could deal with all the crawling and matching, while the FCC processes on the nodes would only have to deal with the actual file processing. This way matching and crawling would only be conducted once.

## Conclusions

FCC streamlines and automates the task of large-scale customized file processing in bioinformatics workflows. It utilizes all available compute resources and commercial licenses efficiently and simplifies configuration and maintenance for life science research facilities.

## Availability and requirements

**Project name:** FGCZ Converter Control (FCC) **Project home page:**http://fgcz-data.uzh.ch/public/fcc/**Operating system(s):** Platform independent for FCC, Mac OS X for graphical user interface **Programming language:** Python 3.2 or higher **Other requirements:** Shared file system for raw files, mechanism for distributing configuration files (e.g Subversion, Dropbox, SSH) **License:** GNU General Public License GPLv3 **Any restrictions to use by non-academics:** none

## Abbreviations

FGCZ: Functional Genomics Center Zurich; FCC: FGCZ Converter Control; ETD: Electron-transfer dissociation; CID: Collision-induced dissociation; EC2: Elastic Compute Cloud; GUI: Graphical User Interface; S3: Amazon Simple Storage Service; SSH: Secure Shell; DNS: Domain Name System.

## Competing interests

The authors declare that they have no competing interests.
